# Unveiling the mechanisms of American ginseng and achyranthes in treatment of primary Sjogren’s syndrome via mtDNA-cGAS-STING pathway insights from network pharmacology, molecular dynamics, and experimental validation

**DOI:** 10.3389/fimmu.2025.1675429

**Published:** 2025-10-03

**Authors:** Haotian Li, Congmin Xia, Yuewei Song, Jin Chen, Yanjun Liu

**Affiliations:** ^1^ Beijing University of Chinese Medicine, Beijing, China; ^2^ Guang’anmen Hospital, China Academy of Chinese Medical Sciences, Beijing, China; ^3^ Institute of Basic Theory for Chinese Medicine, China Academy of Chinese Medical Sciences, Beijing, China

**Keywords:** network pharmacology, molecular docking, cGAS-STING pathway, mtDNA, Sjögren’s syndrome

## Abstract

**Objective:**

With the aim of clarifying the therapeutic mechanisms of the American Ginseng-Achyranthes bidentata (AG&A) herbal pair in primary Sjögren’s syndrome (pSS), this study employs an integrated approach combining network pharmacology, molecular docking, molecular dynamics simulations, and animal experiments.

**Methods:**

Network pharmacology & LC-MS/MS was utilized to identify the active components and potential targets of A&A. Molecular docking and dynamics simulations were performed to evaluate binding affinity and complex stability with key targets. Animal experiments using non-obese diabetic (NOD) mice were conducted to validate symptom improvement by critical active components.

**Results:**

Network pharmacology identified baicalin and quercetin as key active components. Molecular docking revealed strong binding affinities (binding energy ≤ -8.0 kcal/mol) between these compounds and apoptosis-related proteins, BAX and CASP3. Molecular dynamics simulations confirmed the stability of these complexes. Animal experiments demonstrated that baicalin can significantly reduce inflammatory cytokines of IL-18, TNF-α, IFN-α, and IFN-β,CXCL-10 (p < 0.05), decrease mtDNA release, and downregulate cGAS-STING pathway-related proteins including cGAS, STING, CASP3, ZBP1, TBK1, p-STING, p-TBK1, IRF3, p-IRF3 and BAX.

**Conclusion:**

The critical components baicalin and quercetin from AG&A, particularly in aqueous extracts, exhibit therapeutic efficacy against pSS. This study provides experimental evidence for their action mechanism through modulating the mtDNA-cGAS-STING pathway. While highlighting their therapeutic potential, additional *in vivo* and clinical studies are warranted to validate these findings.

## 1 Introduction

Sjögren’s syndrome (SS) is a chronic autoimmune disorder characterized by the involvement of exocrine glands, particularly the salivary and lacrimal glands, leading to common clinical symptoms such as dry mouth and eyes (1). With the prevalence ranging from 0.29% to 0.77%, SS has exhibited an increasing trend observed in recent years (2). This condition predominantly affects women over the age of 40, with a male-to-female incidence ratio of approximately 1:9 (3). Patients with SS are at a higher risk for complications and multisystem damage, with about 30% to 40% experiencing extra-glandular manifestations such as interstitial lung disease and hematologic issues. Additionally, recent studies indicate that SS patients have an elevated risk of developing other tumors compared to the healthy population (4).

Over the years, individuals with SS have been burdened by the disease, leading to a reduced quality of life, yet treatment remains mainly symptomatically and empirically managed, with no biologically or chemically synthesized antirheumatic agents available to modify the disease process (5). Hence, it is urgently needed to identify effective preventive and therapeutic strategies for SS, which holds substantial clinical and socioeconomic significance.

The pathogenesis of SS is multifactorial, involving genetic, environmental, hormonal, and immune elements (6). Although research utilizing genome-wide association studies, epigenomics, transcriptomics, and metabolomics has been extensively conducted (7), the complete mechanisms underlying the disease remain elusive. Current treatments in modern medicine tend to focus on localized symptoms, with artificial fluid replacement therapies commonly used to alleviate dryness. However, these interventions often yield suboptimal results (8), and there are significant challenges in systemic treatment and disease progression management, compounded by safety concerns. Consequently, many patients suffer from persistent clinical symptoms that are not adequately managed.

Hydroxychloroquine (HCQ) is the most frequently prescribed immunomodulatory agent for pSS, primarily acting through interfering Toll-like receptor (TLR) signaling and inhibiting type I interferon (IFN) pathway. Although HCQ demonstrates clinical efficacy in reducing disease activity and is extensively used in clinical treatment, its therapeutic effectiveness in pSS remains incompletely characterized. In addition, current evidence regarding HCQ’s efficacy in pSS patients shows conflicting results. Hence, it is imperative to identify alternative therapeutic agents for treatment (9).

In prior studies, the efficacy of American Ginseng and Achyranthes (AG&A) in treating SS has been highlighted (10, 11). Therefore, the present study aims to explore the mechanisms by which AG&A exerts its therapeutic effects in SS treatment through network pharmacology, LC-MS/MS and molecular docking methodologies, and validate the enrichment results of network pharmacology through *in vivo* experiments in NOD mice.

## 2 Methods

### 2.1 Network pharmacology

#### 2.1.1 Composition of PEs and SS target acquisition

We identified the chemical compositions of the effective pharmacological extracts (PEs) using the methods outlined above. The key active ingredients in the remaining PEs were screened via the Traditional Chinese Medicine Systems Pharmacology (TCMSP) database (12), with oral bioavailability (OB) ≥ 30% and drug-likeness (DL) ≥0.18 the criteria. The SMILES format for these components was collected from the PubChem database (13). Ingredient targets, defined as active ingredients with a probability greater than zero, were obtained from the Swiss Target Prediction database, and any duplicate targets were subsequently removed (14).

#### 2.1.2 Analysis of PE components and SS targets

Targets associated with SS were sourced from the GeneCards (15) and Online Mendelian Inheritance in Man (OMIM)) (16) databases. The intersection of SS-related targets with the identified targets was considered as potential therapeutic targets. Then, we utilized Cytoscape 3.9.1 software to construct the network linking active ingredients, potential targets, and the disease associated with effective PEs for SS treatment.

#### 2.1.3 Construction of the protein–protein interaction network

All targets related to PEs and SS were entered into the STRING database (17) to construct the PPI network. The minimum interaction score was set to above 0.9, with free nodes hidden and all other settings left as default.

#### 2.1.4 GO and KEGG enrichment analysis

To further elucidate the mechanisms of the key targets of the effective PEs in treating SS, these targets were analyzed using the DAVID database (18).

#### 2.1.5 LC-MS/MS analysis of American ginseng and achyranthes components

An aliquot (150 µL) of the herbal formula sample was mixed with 150 µL of 70% methanol (containing internal standards, 2 µg/mL). The mixture was vortexed for 1 min, ultrasonically extracted in an ice-water bath for 60 min, and centrifuged at 12,000 rpm (4°C) for 10 min. The supernatant (200 µL) was transferred to an LC-MS vial and then separated on an ACQUITY UPLC HSS T3 column (100 mm × 2.1 mm, 1.8 µm; Waters) at 45°C for subsequent analysis. Mobile phases consisted of (A) water with 0.1% formic acid and (B) acetonitrile. The gradient elution was: 0–2 min, 5% B; 2–8 min, 5–50% B; 8–10 min, 50–80% B; 10–14 min, 80–100% B; hold until 15 min; return to initial conditions at 15.1 min. The flow rate was set to 0.35 mL/min with a 2-µL injection volume. The analysis was conducted using a Thermo Orbitrap QE HF mass spectrometer equipped with a heated electrospray ionization (HESI) source. Data were acquired in data-dependent acquisition (DDA) mode, alternating between full MS scans (resolution: 60,000; m/z range: 100–1500) and MS/MS scans of the top 8 ions (resolution: 15,000). For the key parameters, raw data were processed using XCMS software (v4.5.1) for peak detection, alignment, and integration. Components were identified by matching against the proprietary LuMet-TCM database (>5,000 herbal standards) with the following criteria: Extracted ion chromatograms (EIC) and annotated MS/MS spectra were generated for verification. Compounds with a total identification score ≥40 were retained for analysis.

### 2.2 Molecular docking and molecular dynamic simulation

#### 2.2.1 Molecular docking

The 3D structure files of the target protein were retrieved from the RCSB Protein Data Bank (19). Structural preparation for molecular docking was performed using AutoDockTools 1.5.7.

#### 2.2.2 Molecular dynamics simulation

The molecular dynamics simulations were conducted utilizing Gromacs (20), under static conditions at a temperature of 310 K and an atmospheric pressure of 1 bar. For preprocessing small molecules, the potential data were incorporated into the topology files of the molecular dynamics system (21). The Amber99sb-ildn force field was employed, with water molecules modeled using the Tip3p water model as the solvent.

### 2.3 Laboratory animal and groups

In this study, NOD mice [a well-established Sjögren’s syndrome animal model documented in scientific literature (22)] were used as the disease model and C57BL/6 mice as the healthy control group(6-10weeks, males, Beijing Beiyou Biotechnology Co., Ltd). We randomly divided them into six groups (n=10 per group), with oral gavage applied for all drug administration.

Specifically, they were Normal control group (C57BL/6 mice), Disease model group (untreated NOD mice), Positive control group (Hydroxychloroquine Sulfate, 6.5 mg/kg/day), Baicalin treatment group (100 mg/kg/day), Quercetin treatment group (100 mg/kg/day), and Herb group (0.1625 g/mL herbal preparation, 16 mL/kg/day). The herbal medicine for oral gavage was prepared by Guang’anmen Hospital in accordance with standard clinical protocols. Animals had free access to feed and water. The room was maintained at a temperature of 23 ± 2°C and a relative humidity of approximately 45%, with a 12-hour light/dark cycle. A one-week acclimatization period preceded the experiment. Water intake was measured gravimetrically over 24h; resting saliva flow was measured: saliva pooled for 1 min was aspirated and volume recorded (mL/min). All therapeutic interventions were administered at equivalent volumes and concentrations through appropriate administration routes. The experimental protocol-maintained consistency in dosage formulation and administration methods across all treatment groups to ensure comparability. Baicalin and quercetin dosages were referenced from the literature and were identified by the additional WB experiment (**Supplementary Material 1**) (23, 24); the herbal dosage was derived from preliminary clinical data (9).

### 2.4 Transmission electron microscopy observation

Three submandibular gland specimens (1mm³) were collected and immersed immediately in 2.5% glutaraldehyde/PBS. They were then rinsed with PBS, post-fixed in osmium tetroxide, dehydrated through ethanol/acetone gradients, and infiltrated with epoxy resin overnight. After that, they were embedded, sectioned stained and finally examined using Transmission electron microscopy (TEM).

### 2.5 Elisa

Tissue lysates or serum samples were analyzed for TNF-α, IFN-β, CXCL10, IL-1β, IL-16, IFN-α, ATP and NAD^+^/NADH concentrations using a commercially available sandwich ELISA kit (RXW202412M, RUXIN BIOTECH, QUANZHOU, CHINA; RXW203063M, RUXIN BIOTECH, QUANZHOU, CHINA; RXW203064M, RUXIN BIOTECH, QUANZHOU, CHINA; RXW203106M, RUXIN BIOTECH, QUANZHOU, CHINA; RXW203124M, RUXIN BIOTECH, QUANZHOU, CHINA; RXW202930M, RUXIN BIOTECH, QUANZHOU, CHINA; INSSH-0089, INSELISA, HUANGSHI, CHINA; INSSH-0028, INSELISA, HUANGSHI, CHINA) according to the manufacturer’s instructions. Absorbance was measured at 450 nm using a microplate reader, and concentrations were interpolated from a standard curve.

### 2.6 Western blotting and RT-PCR

The Western blotting & RT-PCR experimental procedure is consistent with our previous study (25). In the present study, we used: sting: immniway T5488, 1:1000; cGAS: immniway YM8509, 1:5000; TBK1: immniway YM8322, 1:5000; ZBP1: immniway YN2476, 1:1000; BAX: Proteintch 50599-2-IG, 1:50000; Caspase-3: CST 9662S, 1:1000; GAPDH: Servicebio GB11002, 1;1000; IRF3: immniway YM8227, 1:1000;p-IRF3: immniway YP0326, 1:1000; p-STING: immniway YP1518, 1:1000; p-TBK1: immniway YM8674, 1:2000.

Tissues were collected by centrifugation at 600 × g for 5 min, washed with PBS, and repelleted by centrifugation (5 min, 600 × g). The pellet was resuspended in cytosol extraction buffer and incubated on ice for 10 min. Cell disruption was performed using a Dounce tissue grinder, followed by centrifugation at 700 ×g for 10 min to pellet nuclei and intact cells. The supernatant was transferred and subjected to centrifugation at 10,000 g for 30 min. The resulting supernatant was discarded, and the pellet (enriched mitochondria) was resuspended in fresh cytosol extraction buffer, then re-centrifuged at 10,000 g for 30 min to further purify mitochondrial fractions. The final mitochondrial pellet was resuspended in mitochondrial lysis buffer and incubated for 10 min. An enzyme mix was added to the lysate and incubated for 60 min, followed by ethanol addition and a 10-min incubation. Mitochondrial DNA was recovered by centrifugation (5 min), with the resultant pellet retained as purified mtDNA. Primer sequence: CCCCATATTAAACCCGAATGATA; TAGGCTTCGTTGCTTTGAGGTA.

### 2.7 Immunofluorescence

Tissue sections were subjected to immunofluorescence staining using primary antibodies against BAX, cGAS and STING, followed by appropriate Alexa Fluor-conjugated secondary antibodies and DAPI counterstain. Slides were mounted and imaged at 200x magnification using a SlideViewer2.5 scanner, ensuring that tissue filled the field of view and background illumination was consistent across all samples. For each section, three representative fields were quantitatively analyzed. Using image analysis software, the integrated density (IntDen) and positive pixel area (Area) for green, red, pink, and co-localization signals were measured in pixels. The mean fluorescence intensity (MFI) for each channel was calculated as MFI = IntDen/Area.

## 3 Results

### 3.1 Network pharmacology and analysis

**Figure 1:**
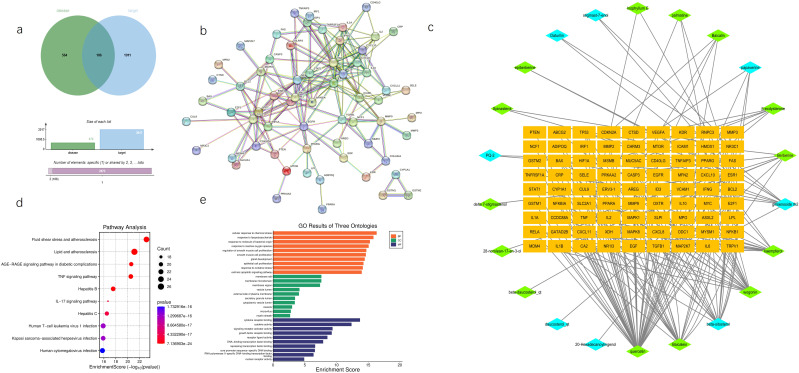
Network pharmacology predicts potential mechanisms of AG&A. **(a)** Disease Target Venn Diagram. Illustrates the overlap between disease-associated targets(bule, contain all databases) and potential therapeutic targets (green, contain all databases) of AG&A. The intersection highlights common targets critical for AG&A’s therapeutic effects. **(b)** Drug-Molecular-Target Network Diagram A bipartite network showing interactions between bioactive compounds and their targets. Orange nodes represent potential therapeutic targets; green nodes denote key compounds from Achyranthes bidentata; blue nodes indicate key compounds from American Ginseng. Node size may reflect compound/target significance. **(c)** Protein-Protein Interaction (PPI) Network Visualizes functional associations between AG&A target proteins. Nodes represent proteins, with edges indicating interactions. Core targets were identified via topology analysis. **(d)** KEGG Pathway Enrichment Analysis Bubble plot displaying enriched GO terms (Biological Process, Molecular Function, Cellular Component) in AG&A targets. X-axis: Statistical significance (-log_10_(p-value); Y-axis: Term name; Bubble size: Gene count; Color:p-value. **(e)** Gene Ontology (GO) Enrichment Analysis Bubble plot showing AG&A target-enriched KEGG pathways. X-axis: Rich Factor; Y-axis: Pathway name. Top pathways are highlighted. **(e)**. KEGG Pathway Enrichment Analysis Bubble plot showing AG&A target-enriched KEGG pathways. X-axis: Rich Factor; Y-axis: Pathway name; Bubble size: Gene count; Color: Statistical significance [-log_10_(p-value)]. Top pathways are highlighted.

**Figure 2:**
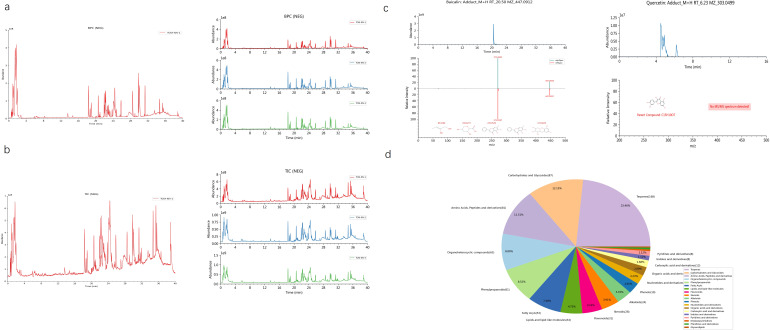
Analysis of baicalin and quercetin by LC-MS/MS. **(a)** Representative base peak chromatogram (BPC) of the AG&A analyzed. The BPC shows the most intense ion current at any retention time during the analysis, providing a chromatographic profile of the most abundant compounds. **(b)** Representative total ion chromatogram (TIC) of the AG&A analyzed. The TIC represents the summed ion current over the entire mass range detected at each retention time, providing an overall chromatographic signature of all detected compounds. **(c)** Mass spectrum of baicalin and quercetin. This spectrum displays the relative abundance of ions at different mass-to-charge ratios (m/z) for the identified compounds, enabling structural identification and confirmation. **(d)** Component Classification Quantity Distribution Diagram. This chart illustrates the relative proportions of different compounds. Each sector of the pie represents a different compound or compound category, with the size of the sector corresponding to the relative quantity of that compound in the sample.

We conducted a comprehensive retrieval of pSS (primary Sjögren’s syndrome) -related targets, yielding a total of 564 targets from the GeneCards database and the OMIM database, with a relevance score greater than 3.41. Following database calibration, the pSS targets identified in the PEs target libraries were imported into the Venny 2.1 tool to generate a Venn diagram, which resulted in an identification of 106 common targets associated with pSS, visually represented in the Venn diagram (**Figure 1a**). The active ingredients from PEs and their corresponding targets were imported into Cytoscape 3.9.1 to construct a comprehensive drug-active ingredient-target network. In this network, nodes represent targets, with node size proportional to the degree of connectivity: larger nodes indicate targets with more biological functions and enhanced significance(**Figure 1b**). Removing irrelevant nodes, the network diagram was generated. Based on LC-MS/MS identification (**Figure 2**, **Supplementary Material 2**), baicalin and quercetin were selected as the active compounds. Through network pharmacology analysis, several key targets were identified (degree>6), including ESR1, PPARG, CASP3, CA2, and BAX. Additionally, the analysis highlighted critical active ingredients (degree>18), such as quercetin, baicalin, beta-sitosterol, and wogonin, which play significant roles in the therapeutic effects of PEs. To elucidate the interactions between PEs and pSS targets, the intersection targets were imported into the STRING database to construct a PPI network, thus highlighting the most critical genes within the PPI network that may significantly influence pSS. The results revealed a network comprising 83 nodes and 196 edges among the 106 overlapping proteins, with an average node degree of 4.77. This indicated a strong correlation among the targets, with a confidence score of ≥ 0.9, suggesting robust interactions within the network (**Figure 1c**). The 106 intersection targets were then submitted to the DAVID database for GO and KEGG enrichment analysis, with the top 10 GO entries across the three categories—BP, MF, and CC— identified and illustrated in **Figure 1d**, and the top 10 KEGG pathways associated with these targets screened and presented in **Figure 1e**. These analyses provided insights into the biological roles and pathways that the common targets may influence in the context of SS.

### 3.2 Molecular docking and molecular dynamics simulations

**Figure 3:**
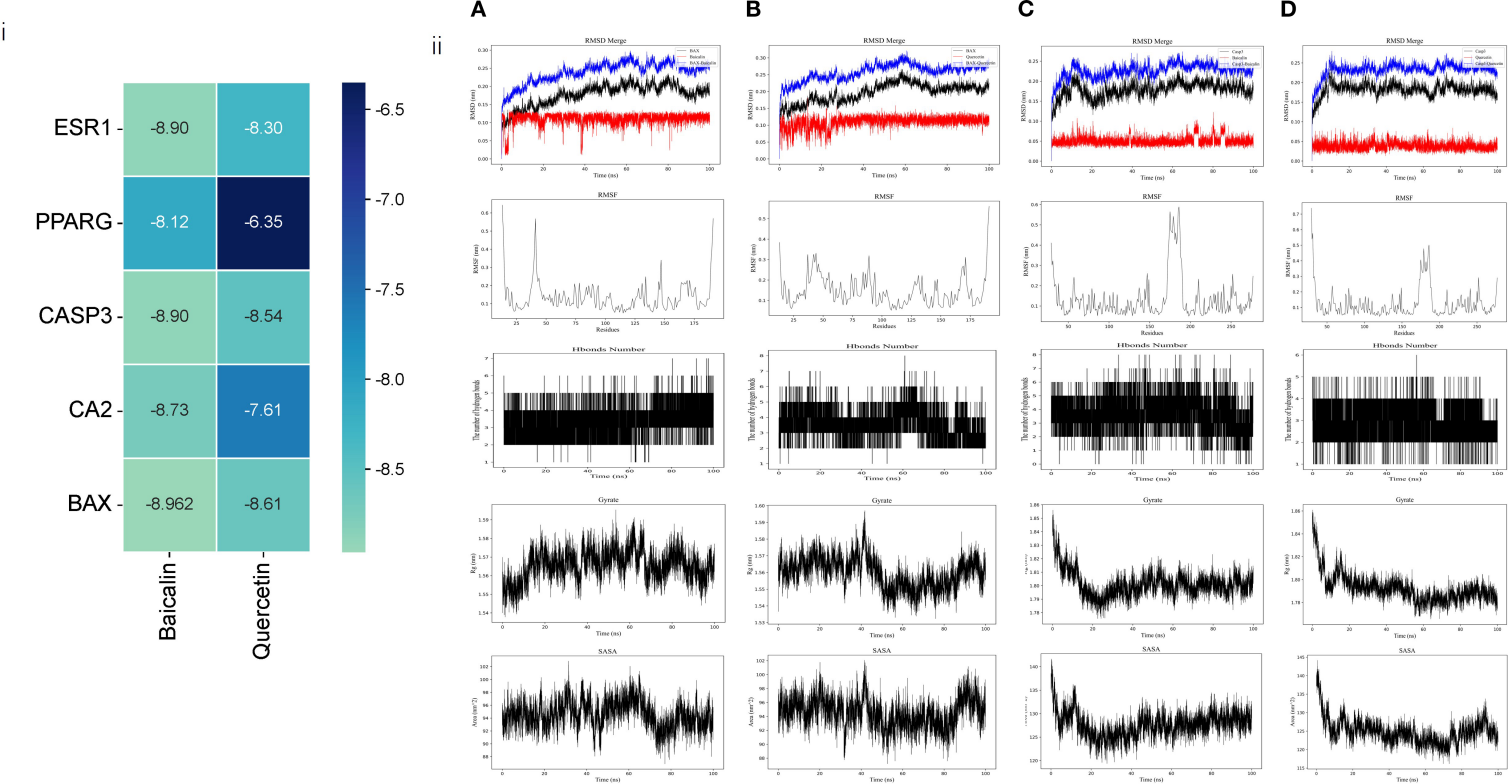
Computational prediction of protein-ligand interactions and molecular dynamics simulations. **(i)** Heatmap depicting predicted binding affinities (kcal/mol) between the two active ingredients (Quercetin, Baicalin) and the five hub targets. Darker green indicates stronger binding (more negative values). **(ii)** Representative snapshots from molecular dynamics simulations of protein-ligand complexes, from top to bottom: RMSD, RMSF, Hbonds Number, Gyrate, SASA: **(A)** BAX in complex with Quercetin **(B)** BAX in complex with Baicalin **(C)** CASP3 in complex with Quercetin **(D)** CASP3 in complex with Baicalin.

Molecular docking was conducted using the key genes and PEs identified in section 3.1 as active ingredients and targets. The docking & molecular dynamics simulations results (**Figure 3**) demonstrated that the selected targets exhibited strong binding affinities with the core active ingredients. This finding further validated the reliability of the network pharmacology analysis, confirming the potential interactions between the active components and their respective targets in the therapeutic context of SS.

While BAX and Caspase-3 (CASP3) were identified as critical targets for AG&A in pSS treatment (binding energy ≤ -8.5 kcal/mol) through network pharmacology and molecular docking, their biological roles extend beyond apoptosis regulation. As a key mediator of mitochondrial outer membrane permeabilization (MOMP), BAX facilitates mitochondrial DNA (mtDNA) release into the cytosol during cellular stress. Concurrently, CASP3—traditionally associated with apoptosis execution—has recently been implicated in modulating innate immune responses via cleavage of cGAS to suppress interferon production (26). Crucially, cytosolic mtDNA acts as a potent ligand for cGAS, initiating the cGAS-STING signaling cascade that drives type I interferon (IFN-I) inflammation—a hallmark of pSS pathogenesis. Given that IFN-I signature correlates with pSS disease severity and glandular dysfunction, we hypothesized that AG&A’s modulation of BAX/CASP3 may functionally intersect with the mtDNA-cGAS-STING axis to resolve immunopathology. Therefore, to elucidate the holistic mechanism underlying AG&A’s efficacy, we experimentally validated its impact on mtDNA leakage, cGAS-STING activation, and downstream inflammatory cascades in submandibular glands.

### 3.3 AG&A ameliorates submandibular gland dysfunction in Sjögren’s syndrome models

**Figure 4:**
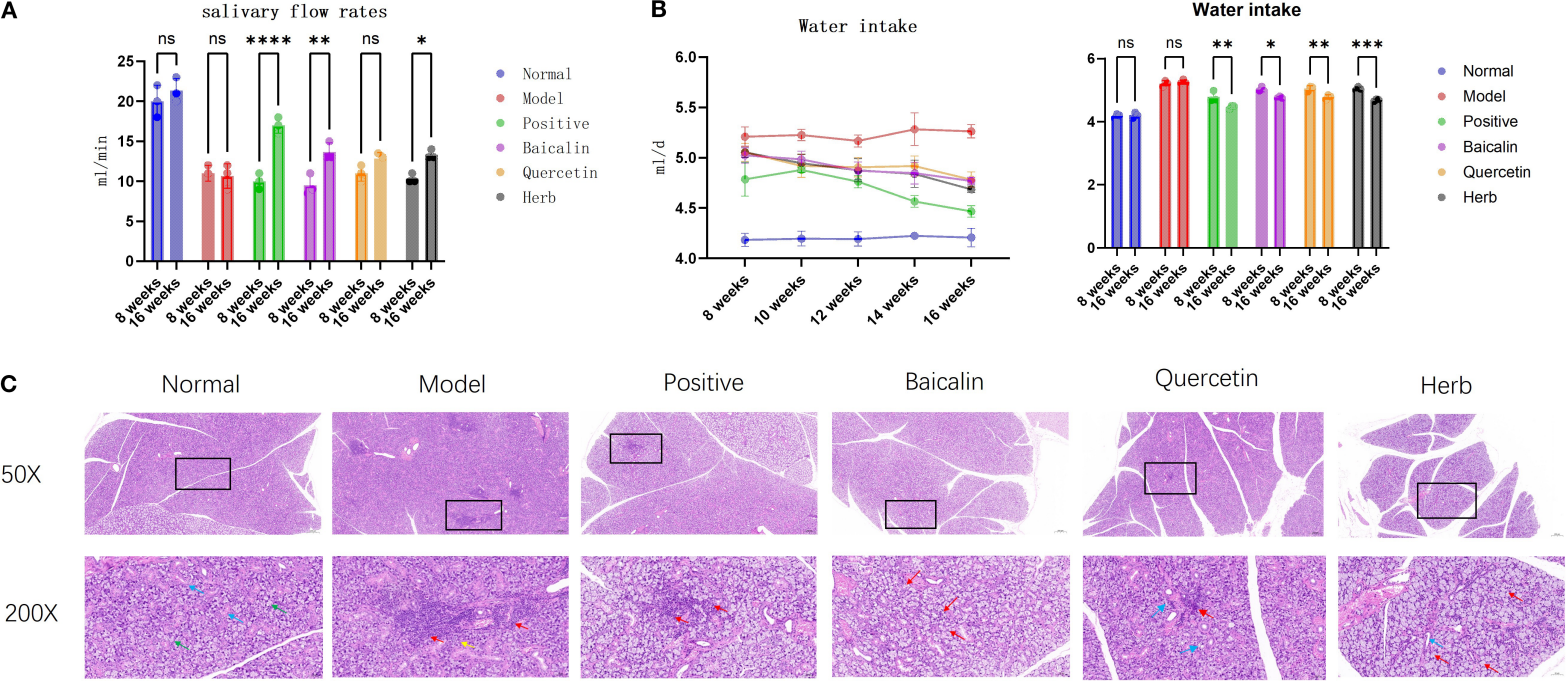
Effects of AG&A and its active compounds ameliorates submandibular gland dysfunction. **(a)** Salivary flow rate. (n=3 per group, Two-way anova was used, Data are presented as mean ± SEM, ns p>0.05, *p < 0.05, **p < 0.01, ***p < 0.001, ****p < 0.0001). **(b)** Daily water intake. (n=3 per group, Two-way anova was used, Data are presented as mean ± SEM, ns p>0.05, *p < 0.05, **p < 0.01, ***p < 0.001, ****p < 0.0001). **(c)** Representative HE stained sections of submandibular. 50X:Scale bar = 200 µm;200X:Scale bar = 50 µm.

AG&A and its key components can significantly improve submandibular gland function in NOD model mice, manifested through enhanced salivary flow rate, reduced water consumption, and histopathological restoration. Specifically, NOD mice exhibited reduction in salivary flow rate compared to normal controls (p<0.001), while pharmacological intervention with AG&A and its active compounds restored salivary secretion to 70-80% of physiological levels (**Figure 4a**). Concomitant with xerostomia, model mice demonstrated increase in daily water intake (p<0.01) (**Figure 4b**). Histopathological examination revealed **Figure 4c**: The normal control group exhibited intact salivary gland architecture with no pathological changes such as acinar degeneration, necrosis, or inflammatory cell infiltration in the stroma. In contrast, the model group showed multifocal, dense inflammatory cell infiltration within the submandibular glands, forming lymphoid follicle-like structures, with mild acinar atrophy observed in surrounding areas. Relative to the model group, all treatment groups demonstrated varying degrees of pathological improvement: The herb group exhibited the most significant amelioration, followed by the baicalin group which presented only minor vacuolar degeneration in acini, and the quercetin group and positive control group showed the mildest changes, with scant inflammatory cell infiltration between acini and slight vacuolar degeneration.

### 3.4 AG&A ameliorates mitochondrial function in submandibular glands of Sjögren’s syndrome model mice

**Figure 5:**
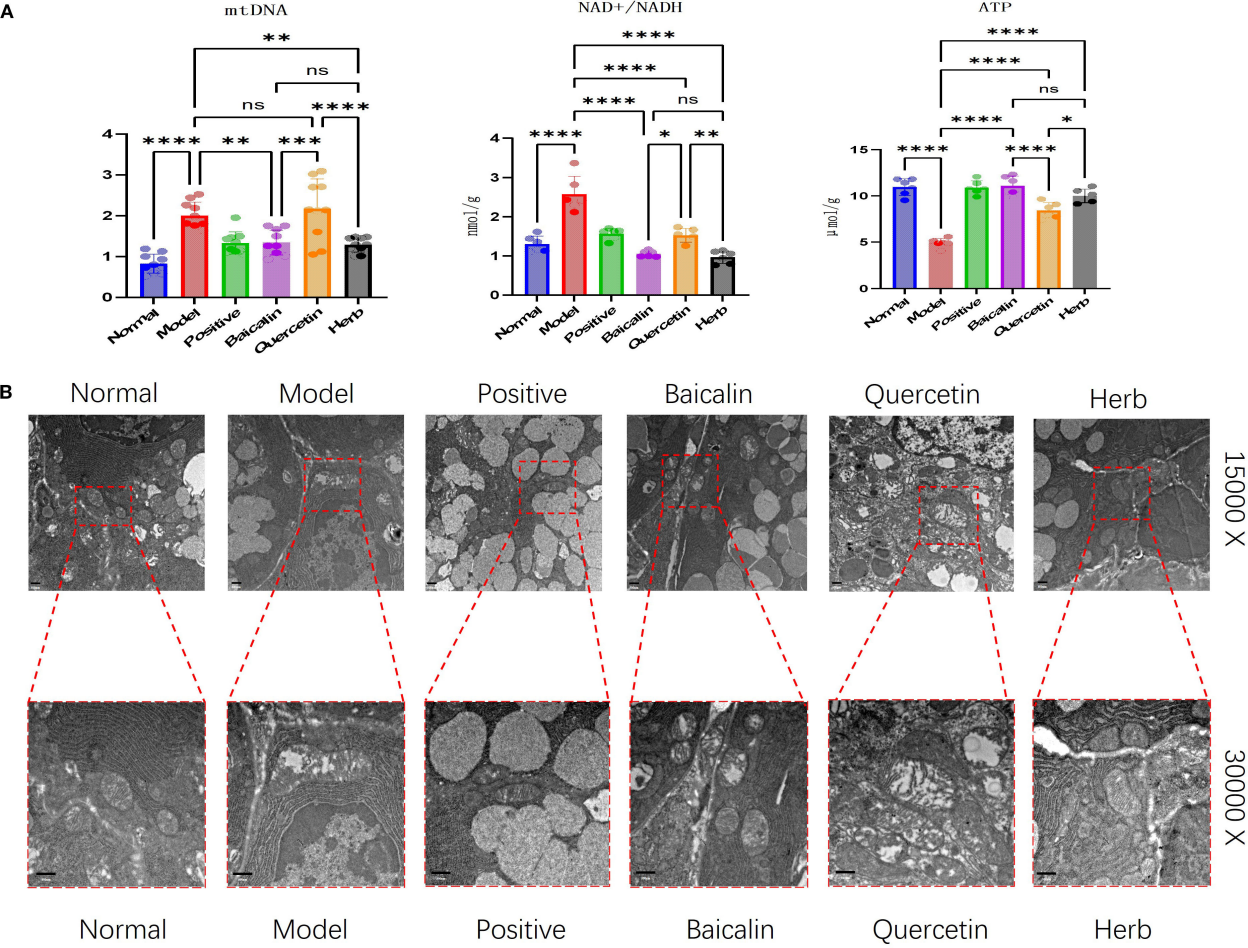
Analysis of AG&A ameliorates mitochondrial function in submandibular gland tissue. **(A)** Concentrations of ATP and NAD^+^/NADH ratio measured by ELISA, and relative mitochondrial DNA (mtDNA) copy number determined by RT-PCR. (ATP and NAD^+^/NADH: n=5 per group, mtDNA:: n=10 per group, one-way anova was used, Data are presented as mean ± SEM. ns p>0.05, *p < 0.05, **p < 0.01, ***p < 0.001, ****p < 0.0001). **(B)** Representative TEM images depicting mitochondrial ultrastructure in submandibular. Scale bars = 500 nm.

Experimental results demonstrated that compared to the normal control group, NOD model mice exhibited significant mitochondrial dysfunction in submandibular glands, characterized by a reduction in ATP synthesis (p<0.01), a 1.8-fold increase in cytoplasmic mtDNA copies, and an abnormal elevation in NAD+/NADH ratio. Trough pharmacological interventions, these abnormalities were effectively reversed: cytoplasmic mtDNA overamplification was suppressed, ATP levels recovered to normal values, and the NAD+/NADH ratio normalized to physiological ranges (**Figure 5A**), indicating substantial restoration of mitochondrial energy metabolism.

These findings were then corroborated through TEM ultrastructural analysis, demonstrated by classical mitochondrial pathologies in acinar cells of SS model mice. Specifically, mitochondria exhibited swelling, and also showed cristae fragmentation or complete loss of mitochondrial structure, as well as vacuolar degeneration. Post treatment, all groups except the quercetin group demonstrated marked morphological recovery, with the most pronounced restoration observed in the herb group (**Figure 5B**).

### 3.5 AG&A corrects mitochondrial dysfunction via cGAS/STING signaling to ameliorate immune microenvironment in submandibular glands

Mechanistically, mitochondrial dysfunction in submandibular glands of NOD model mice triggers pathological leakage of mtDNA into the cytosol through mitochondrial permeability transition pores, thereby activating the cytosolic DNA-sensing cGAS-STING signaling axis. This activation initiates a proinflammatory cascade characterized by upregulated type I interferons (IFN-α/β) and inflammasome-related mediators.

**Figure 6:**
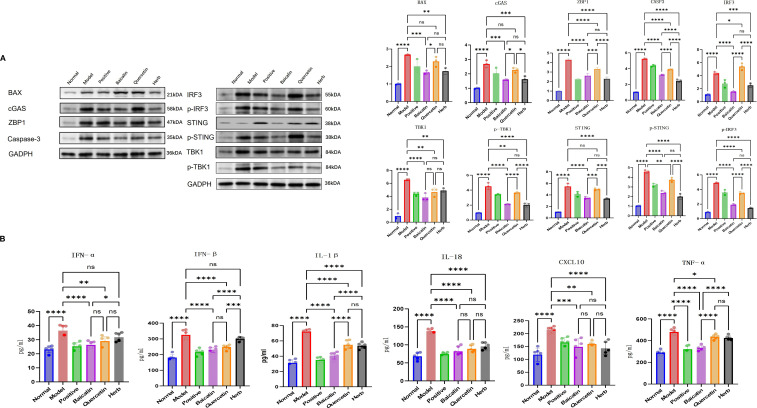
AG&A corrects mitochondrial dysfunction via cGAS-STING signaling to ameliorate immune microenvironment. **(A)** Western blot analysis of BAX, cGAS, STING, p-STING, ZBP1, TBK1, p-TBK1, IRF3, p-IRF3 and CASP3 protein levels. Densitometric quantification of protein bands normalized to loading control is presented as mean ± SEM. (n=3 per group, one-way anova was used, ns p>0.05, *p < 0.05, **p < 0.01, ***p < 0.001, ****p < 0.0001). **(B)** Concentrations of IFN-α, IFN-β, CXCL10, IL-1β, IL-16, and TNF-α in serum measured by ELISA. Data are presented as mean ± SEM (n = 5 per group, one-way anova was used,ns p>0.05, *p < 0.05, **p < 0.01, ***p < 0.001, ****p < 0.0001).

**Figure 7:**
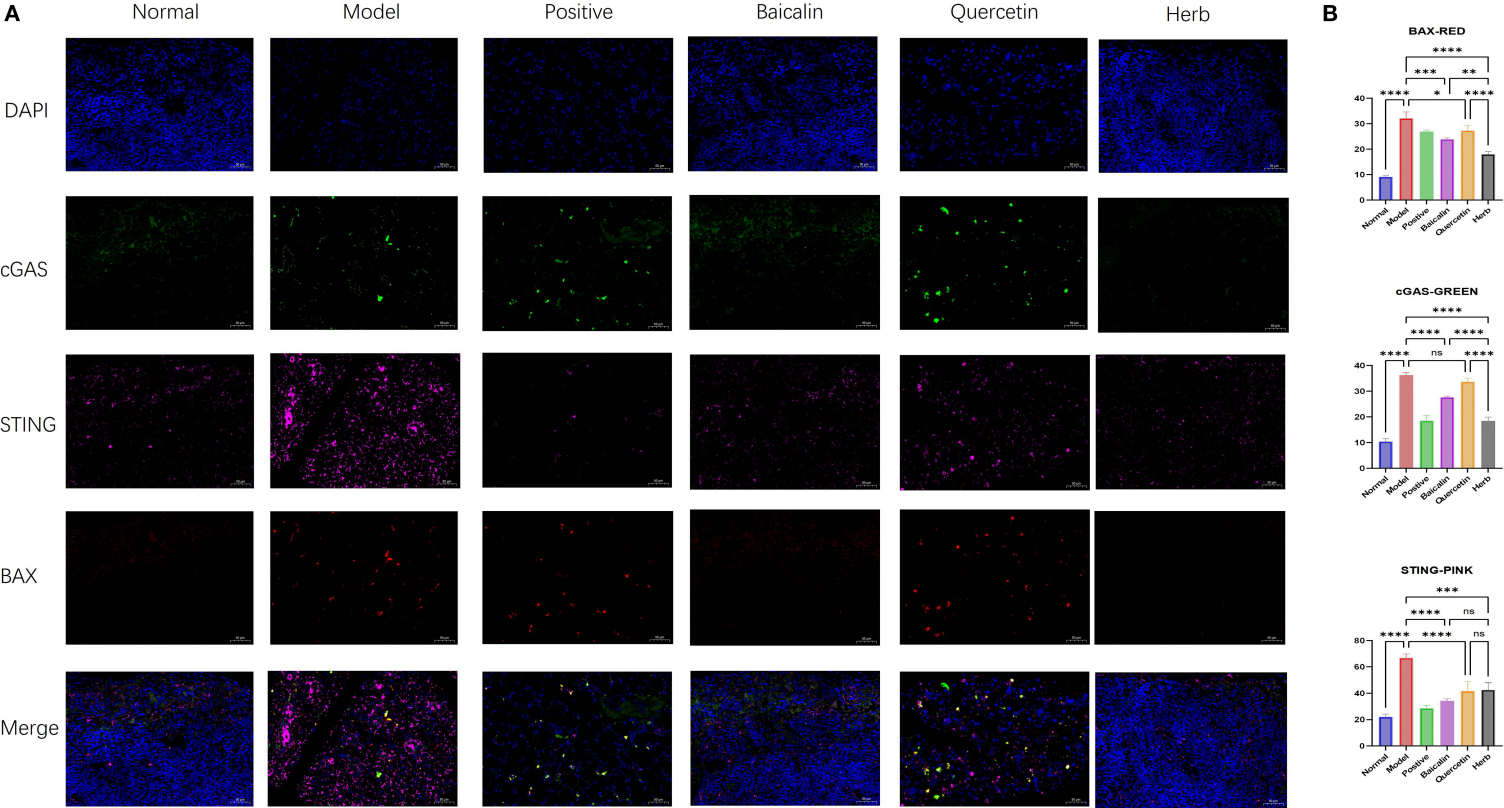
Immunofluorescence analysis of BAX, cGAS, and STING expression in submandibular gland tissue. **(A)** Representative immunofluorescence images showing localization of (red) BAX, (green) cGAS, and (pink) STING. Nuclei were counterstained with DAPI (blue). Scale bar = 50 µm. **(B)** Quantitative analysis of mean fluorescence intensity (MFI) for BAX, cGAS, and STING. MFI was calculated as Integrated Density (IntDen)/Area within regions of interest. Data are presented as mean ± SEM (n = 3 per group; one-way anova was used, Scale bars = 50 µm; ns p>0.05, *p < 0.05, **p < 0.01, ***p < 0.001, ****p < 0.0001).

Western blot analysis confirmed significant dysregulation of key pathway components: Model mice exhibited marked upregulation of BAX, cGAS, STING, ZBP1, TBK1, p-STING, IRF3, p-IRF3, p-TBK1 and CASP3 compared to normal controls (P<0.05 for all). This hyperactivation was differentially suppressed by pharmacological interventions: Baicalin & herb Group showed most pronounced reductions (P<0.05) (**Figures 6**, **7**) across all targets, and Quercetin Group demonstrated marginal efficacy (P>0.05). Notably, the therapeutic hierarchy (AG&A/baicalin > positive control > quercetin) aligned precisely with functional recovery patterns observed in salivary flow restoration & histopathological improvements (Section 3.3) and mitochondrial protection (Section 3.4). These biochemical alterations substantiate AG&A’s dual mechanism: protecting mitochondria to prevent mtDNA leakage and modulating cGAS/STING axis to resolve interferon-driven inflammation, thus synergistically restoring glandular immune homeostasis.

## 4 Discussion

It is recognized that the treatment with traditional Chinese medicine (TCM) is effective for SS. Clinical treatment has shown that the application of TCM in SS management can significantly alleviate symptoms, slow disease progression, and maintain safety. Therefore, a deeper exploration of its therapeutic mechanisms was conducted to. provide a new foundation for the further clinical application of TCM in SS treatment.

Previous studies have indicated that American ginseng can counteract mitochondrial dysfunction (MDF) and apoptosis induced by oxidative and inflammatory damage through inhibiting calcium (Ca²^+^) influx, thereby maintaining cellular homeostasis (27). Additionally, it has been proved that achyranthes bidentata possesses antioxidant properties, thus being helpful to protect mitochondrial ultrastructure (28). Recent research shows that the cGAS-STING pathway likely contributes to the pathogenesis of autoimmune diseases, including desiccation syndrome. Furthermore, the abnormal leakage of mtDNA due to mitochondrial damage has been recognized as a critical mechanism for activating the cGAS-STING pathway. Thus, the present study explored how AG&A regulates the mtDNA-cGAS-STING signaling axis, aiming to correct MDF and regulate the metabolic reprogramming of immune cells to prevent immunoinflammatory alterations. This regulation may further impact the immune microenvironment of the submandibular gland, which supports the notion that Bidentate Achyranthes and American Ginseng represent an effective therapeutic intervention for SS, highlighting its significant research value.

The superior therapeutic outcomes of the whole herb AG&A formulation compared to high-dose baicalin or quercetin monotherapy—particularly in mitochondrial ultrastructural restoration and cGAS-STING pathway suppression—indicate multifaceted synergism. This likely arises from pharmacokinetic enhancement, target diversification, and pathway crosstalk amplification. In contrast, quercetin’s marginal efficacy stems from extensive first-pass metabolism, and context-dependent activity, and its weak CASP3 binding further limits apoptosis regulation and mitochondrial rescue.

As baicalin and quercetin are flavonoids, we included virtual screening data for other AG&A flavonoids (**Supplementary Material 3**). Notably, our results revealed two constituents within the mixture, Trifolirhizin and Rottlerin, which demonstrated superior binding affinity compared to Baicalin (previously identified by Network Pharmacology). Besides, Wang, D (29). proved that Rottlerin can inhibit cGAS-STING signaling pathway in mice. Furthermore, both Trifolirhizin and Rottlerin have been independently documented in other studies as potent inhibitors of mitophagy (30). This strongly suggests that these two compounds may be the key synergistic substances responsible for the enhanced efficacy observed in the herb, compared with baicalin alone.

The submandibular gland is the organ most commonly affected in SS, with an abnormal immune microenvironment in the gland being the primary cause of dry mouth due to decreased secretory function. In this altered immune microenvironment, the epithelial cells of the submandibular gland can directly activate CD4+ T cells by expressing major histocompatibility complex class II (MHC-II), co-stimulatory molecules (CD80 and CD86), and intercellular adhesion molecule-1 (ICAM-1) under inflammatory stimuli. Furthermore, these activated epithelial cells enhance the immune response through elevated expression of chemokines such as CXCL12 and CXCL13, as well as pro-inflammatory cytokines including IL-1, IL-6, and BAF, thereby driving the inflammatory response. In autoimmune diseases, an uncontrolled inflammatory response often prompt the immune system to attack and destroy healthy cells and tissues, a cascade commonly associated with MDF (31). Research indicates that the pathogenesis of SS is linked to changes in the immune microenvironment of the submandibular gland, which closely correlates with MDF and immune inflammation (32). MDF will result in the release of damage-associated molecular patterns (DAMPs), including mitochondrial DNA (mtDNA), into the cytoplasm. This release then further activates pattern-recognition receptors (PRRs), thus stimulating signaling pathways, including the cGAS/STING pathway. DAMPs can activate PRRs and initiate signaling cascades such as the NLRP3 inflammasome, TLR9, and ZBP1, ultimately triggering an inflammatory immune response.

Recent studies demonstrate that abnormal leakage of mtDNA can activate several key innate immune response pathways, particularly the cGAS-STING signaling pathway, whose activators play crucial roles in cellular immunity. Prolonged immune dysregulation can lead to various pathological immune responses, disrupting the immune microenvironment and the body’s homeostasis under mitochondrial stress conditions. Specifically, when mitochondria are stressed, mtDNA may be released into the cytoplasm through mechanisms such as BAX/BAK-dependent mitochondrial outer membrane permeabilization (MOMP) or the mitochondrial permeability transition pore (mPTP). Once in the cytoplasm, mtDNA readily binds to cGAS, activating the downstream STING signaling pathway, which generates type I interferon (IFN) and triggers inflammatory responses. Under normal physiological conditions, caspases effectively inhibit mtDNA-induced cGAS-STING activation by cleaving cGAS and IRF3, thereby preventing the production of IFN and subsequent inflammatory responses. However, during immune inflammation, apoptosis-mediated caspases may lose their enzymatic activity, leading to an intensified IFN response. This abnormal activation of the mtDNA-cGAS-STING signaling axis results in the overexpression of IFN and various inflammation-related genes (33). Moreover, the activated ZBP1 interacts with TBK1, thus promoting the phosphorylation of IRF3 and further enhancing the production of type I IFN. Also, ZBP1 can activate the NF-kB signaling pathway through TBK1. This intricate interplay underscores the significance of the mtDNA-cGAS-STING signaling axis in mediating immune responses and highlights potential therapeutic targets for managing autoimmune conditions like SS.

## 5 Conclusion

Novel technologies and methods have brought unprecedented opportunities for the research of traditional medicine (34–36). In this study, we identified AG&A as a potential effective herbal combination based on the preliminary clinical data. Through LC-MS/MS and network pharmacology analysis, we determined the potential active components and targets, and thus identified baicalin and quercetin as potential active substances, and BAX and CASP3 as potential effective targets. The promising binding performance of these small molecules with the potential targets was confirmed by molecular docking and molecular dynamics simulations. Additionally, we selected the most relevant cGAS-STING pathway for *in vivo* animal experiments, providing multi-level evidence of baicalin’s therapeutic efficacy, including histopathological examination, mitochondrial function assessment, pathway activation, and local immune microenvironment analysis. Notably, this study also found that the efficacy of the herbal mixture was comparable to high dose baicalin, and even better in certain aspects, such as mitochondrial function protection. Although further exploration was limited by space constraints, this suggests that other substances in the AG&A combination may act as potential mitochondrial protectors or exhibit broader drug effects. Besides, this study focuses only on small clinical doses, necessitating further research on different dosages.

## Data Availability

The original contributions presented in the study are included in the article/**Supplementary Material**. Further inquiries can be directed to the corresponding author.
