# Supplementary material for: Unveiling the mechanisms of American ginseng and achyranthes in treatment of primary Sjogren’s syndrome via mtDNA-cGAS-STING pathway insights from network pharmacology, molecular dynamics, and experimental validation
Source: Front Immunol. 2025 Oct 3;16:1675429. doi: 10.3389/fimmu.2025.1675429 (PMC12531212; doi:10.3389/fimmu.2025.1675429)
Supplement: Supplementary file 1 [file DataSheet1.docx]

Supplementary Material

# Supplementary Data

S1 WB results of key protein（Cgas&sting） expression at different doses

The selection of doses for baicalin (100 mg/kg/day) and quercetin (100 mg/kg/day) administered to the NOD mice was based on well-established precedent within the relevant pharmacological literature investigating these compounds in murine models of inflammation and autoimmunity (e.g., Chang L, Kong A, Guo Y, et al. Quercetin ameliorates salivary gland apoptosis and inflammation in primary Sjögren's syndrome through regulation of the leptin/OB-R signaling. Drug Dev Res. 2022;83(6):1351-1361. doi:10.1002/ddr.21964;Shen, B., Zhang, H., Zhu, Z., Ling, Z., Zeng, F., Wang, Y., & Wang, J. (2023). Miao, L., Zhang, X., Zhang, H., Cheong, M. S., Chen, X., Farag, M. A., Cheang, W. S., & Xiao, J. (2024). Baicalin ameliorates insulin resistance and regulates hepatic glucose metabolism via activating insulin signaling pathway in obese pre-diabetic mice. Phytomedicine : international journal of phytotherapy and phytopharmacology, 124, 155296. https://doi.org/10.1016/j.phymed.2023.155296). There is clear literature support for the treatment of Sjogren's syndrome with quercetin, but there is relatively little literature on the treatment of Sjogren's syndrome with baicalein or the activation of cGAS STING. Although we did not find any literature that directly treats or activates baicalin similar to our study, we referred to articles using the same animal model as our study to develop the preliminary optimal dose. Therefore, we conducted preliminary experiments on baicalin. To empirically validate the appropriateness of these doses for our specific model and pathway endpoints, we conducted a preliminary dose-ranging study (n=3 per group). This pre-experiment tested low, medium, and high doses relative to the literature range. Crucially, analysis of key cGAS-STING pathway components (cGAS, STING) in these pre-experimental groups revealed no statistically significant dose-dependent effects on protein expression levels within the tested range. Consequently, we proceeded with the established and commonly cited dose of 100 mg/kg/day for both compounds in the definitive study. The omission of detailed pre-experiment dose-response data from the main manuscript stemmed primarily from technical challenges encountered during the initial Western blotting (WB) phase for cGAS. Our initial attempts utilized a polyclonal antibody, which resulted in substantial non-specific bands, complicating the interpretation of cGAS expression specifically in the context of subtle dose variations. To ensure transparency and address potential questions regarding dose selection robustness, we have now included the complete WB images and corresponding densitometric analysis from these pre-experimental dose-ranging groups in the Supplementary Materials section. This supplementary data confirms the absence of a significant dose effect on cGAS and STING expression across the tested doses and provides the technical context for our dose selection rationale.


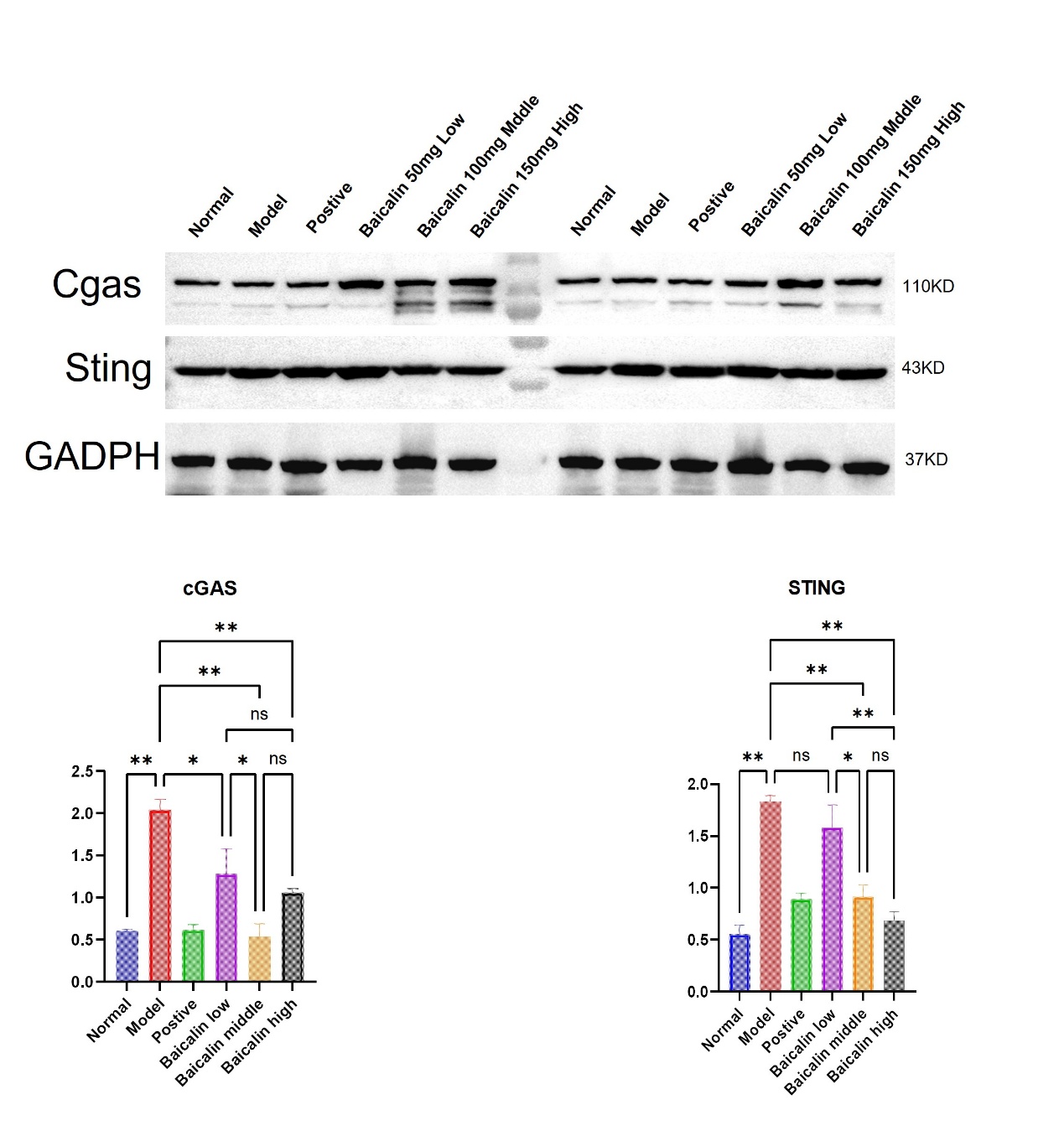


**Supplementary Figure 1.** **WB results of key protein（cGAS&STING） expression** **at different doses**

The wb result of cGAS&STING at different doses

The analyze of the wb result Data are presented as mean ± SEM (n = 3 per group, one-way anova was used ,ns p>0.05, *p < 0.05, **p < 0.01, ***p < 0.001, ****p < 0.0001).
